# The Use of CBCT in Evaluating the Health and Pathology of the Maxillary Sinus

**DOI:** 10.3390/diagnostics12112819

**Published:** 2022-11-16

**Authors:** Andy Wai Kan Yeung, Kuo Feng Hung, Dion Tik Shun Li, Yiu Yan Leung

**Affiliations:** 1 Oral and Maxillofacial Radiology, Applied Oral Sciences and Community Dental Care, Faculty of Dentistry, The University of Hong Kong, Hong Kong SAR, China; 2 Oral and Maxillofacial Surgery, Faculty of Dentistry, The University of Hong Kong, Hong Kong SAR, China

**Keywords:** cone beam computed tomography, CBCT, maxillary sinus, Schneiderian membrane, health, pathology, sinus floor elevation

## Abstract

The use of cone-beam computed tomography (CBCT) has been increasing in dental practice. This narrative review summarized the relevance and utilizations of CBCT to visualize anatomical structures of the maxillary sinus and common pathologies found in the maxillary sinus. The detection/visualization rate, the location and the morphometric characteristics were described. For sinus anatomy, the reviewed features included the posterior superior alveolar artery, sinus pneumatization, sinus hypoplasia, sinus septa, and primary and accessory sinus ostia. For pathology, the following items were reviewed: membrane thickening associated with periapical lesions/periodontal lesions, mucous retention cyst, and antrolith. The visualization and assessment of the maxillary sinus is very important prior to procedures that take place in close proximity with the sinus floor, such as tooth extraction, implant insertion, and sinus floor elevation. Some sinus pathologies may be associated with odontogenic lesions, such as periapical diseases and periodontal bone loss.

## 1. Introduction

The maxillary sinuses are a pair of large air-filled cavities located superior to the posterior part of the dentoalveolar region of the maxilla. The maxillary sinus has the shape of a pyramid and is the largest among the four paranasal sinuses. It drains into the middle meatus of the nasal cavity. It has three recesses, the alveolar process, the zygomatic recess, and the infraorbital recess. Due to the proximity of the sinus floor with the alveolar ridge, the maxillary sinus is of high relevance to multiple dental procedures, ranging from tooth extraction to implant insertion. When the sinus condition should be considered during diagnosis and treatment planning, the cone beam computed tomography (CBCT) is the optimal choice among the common imaging modalities, as it allows a 3D visualization of the structure either entirely or partially, and without the limitation of superimposition with other structures as with 2D imaging. It was reported that 10–14% of the population in Europe and the United States suffered from chronic sinusitis, which could be rhinosinusal or odontogenic [1]. Subsequently, pathologies of the paranasal sinuses were common incidental findings from CBCT imaging [2]. For example, panoramic radiography could not accurately demonstrate root proximity with the sinus floor when there was root contact with the sinus floor on the buccal or palatal side [3]. In these circumstances, panoramic radiography tended to overestimate the extent of root protrusion into the sinus [4], and could make the root appear to be in the sinus [5]. Similarly, panoramic radiography showed inferior performance in detecting sinus membrane thickening [6], sinus septum [7], or oroantral communication after tooth extraction [8]. On the other hand, anatomical variations of the maxillary sinus could sometimes “false positively” presented as apparent odontogenic lesions such as radicular cysts associated with maxillary teeth on a panoramic radiograph, which required further evaluation by CBCT [9].

Dental CBCT machines were developed and commercialized near the end of the 1990s and since then they have been heavily used in dental implant research to assess the condition of the maxillary sinus [10,11]. In fact, the European Academy of Dentomaxillofacial Radiology (EADMFR) advocated a minimum level and core content for training dentists who were involved in CBCT imaging and interpretation for general dentists [12]. In Level 1, a general dentist should be able to use CBCT machines, analyse normal anatomical structures of the teeth, jaws and facial skeleton in CBCT images, and recognize both anatomy and disease of the teeth and supporting structures in CBCT images [12]. In Level 2, a general dentist should be able to differentiate between findings indicating normal anatomical structures from those of diseased teeth, jaws and facial skeleton [12]. Therefore, the correct interpretation of the status of the maxillary sinus is one important aspect in reading CBCT images, as the sinus is frequently in close proximity of the root apices of the maxillary posterior teeth.

In clinical practice, one of the most frequent indications for dental CBCT imaging was the dimensional and health assessment of the alveolar ridge and maxillary sinus for implant planning and sinus lift procedures [13], including the assessment of the bone graft volume required [14]. Indeed, several authorities have advocated the use of CBCT for pre-operative evaluation of the implant planning sites [15,16,17]. Besides implant planning, evaluating the health status of the maxillary sinuses during the diagnostic and treatment planning procedures has also been suggested in other scenarios, such as orthodontics [18], pre-operative assessment of teeth undergoing apical surgery [19], and evaluation of cystic and neoplastic lesions in the maxillary region especially if the posterior region was involved [20].

As mentioned above, a CBCT scan produces a 3D image volume, and hence its radiation dose is usually higher than a plain radiograph such as a panoramic or a periapical radiograph. Some clinicians may thus minimize the use of CBCT and subsequently be unfamiliar with the advantages of this modality over plain radiography in evaluating the maxillary sinus. With the versatility of a CBCT machine, multiple parameters ranging from the spatial resolution, peak voltage to the number of projections can be adjusted to optimize the radiation dose emitted, such that the principle of “as low as reasonably achievable” (ALARA) has gone through a long history of evolution [21], with some expert opinions that advocated to rename it to “as low as diagnostically acceptable” (ALADA) [22] and, more recently, “as low as diagnostically acceptable being indication-oriented and patient-specific” (ALADAIP) [23]. One quality assurance measure to optimize the radiation dose is to establish institutional or national dose reference levels (DRLs) for each indication of CBCT examinations. For instance, it was suggested that the DRL for a CBCT examination to evaluate maxillary sinus pathology could be in the range of 520–1150 mGy × cm^2^ [24]. Another measure is to enhance user training to minimize the chance of a retake due to inadequate diagnostic value of the original scan. It was recently reported that CBCT retake rate was approximately 2.8% across studies [25], and that inadequate visualization of the maxillary sinus was one of the commonest reasons for a retake [26].

The anatomical variants of the maxillary sinus are mainly sinus septations, presence of accessory sinus ostium, and sinus hypoplasia, among the others [27]. Meanwhile, its pathologies can generally be classified into inflammatory, iatrogenic, traumatic, neoplastic, odontogenic, congenital and bone-related [28]. This narrative review aimed to provide an overview of the findings from published CBCT studies that evaluated the anatomy and pathology of the maxillary sinus.

## 2. Anatomy

### 2.1. Posterior Superior Alveolar Artery (PSAA) and Lateral Wall Thickness

**Figure 1:**
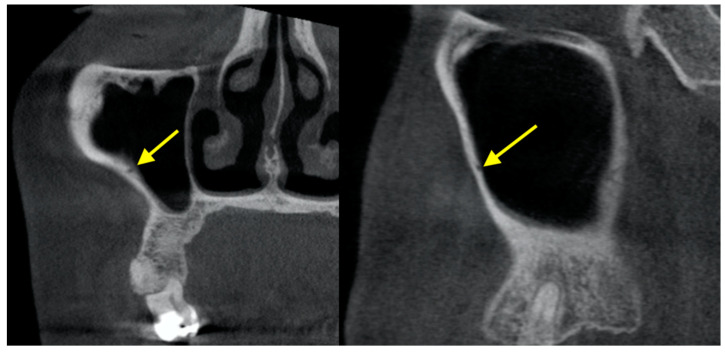
Posterior superior alveolar artery shown in CBCT.

The PSAA is a branch of the maxillary artery that runs along and supplies the lateral wall of the maxillary sinus and the overlying Schneiderian membrane (Figure 1). It should be carefully considered during sinus grafting procedures as there may be a potential risk of profound bleeding [29]. Severe bleeding after damaging PSAA during sinus floor elevation with transcrestal technique has been documented [30]. The detection/visualization rate of PSAA on CBCT scans ranged from around 48.6 to 91.6% [31,32,33,34,35,36]. PSAA could be mostly found below the sinus membrane (13–63.8%) or intraosseous (28.5–71.1%), but less frequent at the outer cortex of the lateral wall of the sinus (5.2–9.9%) [31,32,33,34,35,36]. The apparent absence of PSAA in some CBCT scans could be due to its small size or lying immediately on either side of the lateral wall of the sinus without forming bone indentation [36]. For a meta-analysis on the prevalence of PSAA in a pooled collection of CBCT and CT studies, readers should refer to [37].

Meanwhile, the lateral wall thickness of the maxillary sinus might be critically assessed by CBCT imaging during lateral sinus elevation procedures. A retrospective study of 209 patients with CBCT scans found that the overall mean lateral wall thickness was 1.6 ± 0.8 mm at both 4 mm and 6 mm apical (superior) to the sinus floor [38]. However, cases with membrane perforation during operation had a mean lateral wall thickness of 2.4 ± 0.6 mm at both 4 mm and 6 mm apical to the sinus floor, compared to 1.2 ± 0.4 mm for the non-perforation cases [38]. The lateral wall of the maxillary sinus was also found to be thicker in partial edentulism cases (1.3 ± 0.3 mm) compared to complete edentulism cases (1.0 ± 0.3 mm), and positively correlated with residual ridge height [39]. Considering the mesiodistal direction, the lateral wall thickness at 30–40 mm inferior to the orbital floor showed a gradual reduction from the canine region towards the second molar region [40]. There was a positive correlation between the diameter of PSAA and the thickness of the lateral wall of the maxillary sinus [41].

### 2.2. Pneumatization

Maxillary sinus pneumatization does not seem to have a precise definition. In general, it refers to the maxillary sinus extension into a particular anatomical structure, such as the alveolar ridge, maxillary tuberosity, and palate [42]. For instance, alveolar pneumatization was considered present if the maxillary sinus floor was coronal to the apex of one of the roots of the posterior teeth [43]. Its overall prevalence from CBCT studies ranged from 23.5% to 83.2% [42,43]. It was more common among patients aged between 18 and 34 years (66.4%), followed by those aged between 35–59 years (36.8%) and those aged 60 years or above (22.3%) [44]. It was believed that the alveolar pneumatization was the most common variant, involved in as many as 100% of patients with sinus pneumatization, followed by the palate/palatal process (23.6%), maxillary tuberosity (22%), and anterior region (5.2%) [42]. Alveolar pneumatization tended to be most severe between the first and second molars (13.8–14.0 mm), followed by between the second and third molars (13.22 mm) and between the second premolar and first molar (9.1–9.2 mm) [45]. Similarly, localized sinus pneumatization following tooth loss was more likely to occur at the first molar (37.9%), compared to the second molar (12.1%) [43]. After minimally traumatic molar extraction, alveolar ridge preservation with synthetic nanohydroxyapatite was found to associate with 0.7 mm sinus pneumatization, slightly smaller than the 1.0 mm from unassisted socket healing [46]. The difference was statistically insignificant [46]. For palatal pneumatization, most of them (>99%) did not extend medially to exceed one-half of the width of the nasal floor [47].

### 2.3. Hypoplasia

In contrast to sinus pneumatization, sinus hypoplasia leads to reduced sinus cavity volume. At first glance, this seems to be a favourable condition given the ample distance between the dentoalveolar region and the sinus floor. However, the presence of sinus hypoplasia in CBCT images was found to significantly increase the prevalence of sinus mucosal thickening and blocked ostium (68.3% vs. 25.6%) [48]. The prevalence of sinus hypoplasia was reported to be around 0.2–50% [42,48,49]. Most of the cases belonged to Type I (69.5%; mild hypoplasia, normal uncinate process, and patent infundibular tract) or Type II (25.6%; moderate hypoplasia, hypoplastic uncinate process, and ill-defined or absent infundibular tract). Type III accounted for a minority (4.9%; severe sinus hypoplasia and absence of uncinate process) [48]. In rare circumstances, aplasia might be found from CBCT imaging as an incidental finding [50]. Apart from sinus health, sinus hypoplasia did not seem to affect the health or functions of dentoalveolar structures, such as canting of the maxillary occlusal plane, open bite, or mandibular asymmetry [51].

### 2.4. Sinus Augmentation

In implant cases where the severely resorbed alveolar bone ridge was unable to accommodate an implant, sinus augmentation procedure should be performed to increase the amount of bone inferior to the maxillary sinus floor. Recent CBCT studies found that such bone graft volume decreased by an average of 25% after 4.7–6 months of healing [52,53]. From 6 months after grafting until 3 years after grafting, the graft volume could be reduced by an average of 27.4–39.3% [53,54]. However, one CBCT study found no such significant reduction in the graft volume 4–7 months after the surgery [55]. Apart from bone volume, the bone mineral density is also an important factor for successful implantation. The gray level measured at the implant sites in CBCT images was found to have a good correlation to the actual mineralized material content computed by microradiography of the bone core biopsies taken at the implant sites [56]. Additionally, the gray level of sites grated with bovine bone materials remained stable at 4–7 months of follow-up [55].

### 2.5. Septa

**Figure 2:**
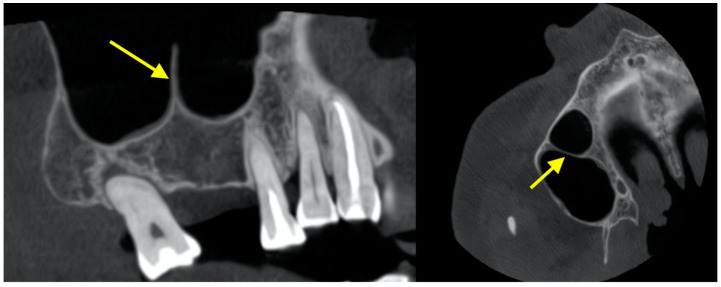
Nasal septum on the sinus floor.

The presence of sinus septa at the sinus floor and lateral wall of the sinus may hinder sinus lift procedures (Figure 2). The prevalence of sinus septa was around 27.1–59.7% [42,57,58,59,60]. Septa could be orientated in a coronal direction (61.8–79.2%), axial direction (4.9–22.3%) or sagittal directions (3.6–24.5%) [57,58,59,61]. As many as 4 septa within one sinus have been reported [57,58]. In the caudocranial direction, septa was most prevalent in the sinus floor (58.6%), followed by roof (33.1%), anterior wall (6.7%), posterior wall (1.2%), and lateral/medial wall (0.4%) [57]. In the anteroposterior direction, septa was most prevalent in the region of the 1st and 2nd molars (37.2–69.1%), followed by the region of the third molar (18.6–33.0%) and the region of the canines and premolars (12.2–29.8%) [59,60].

### 2.6. Primary and Accessory Ostia

**Figure 3:**
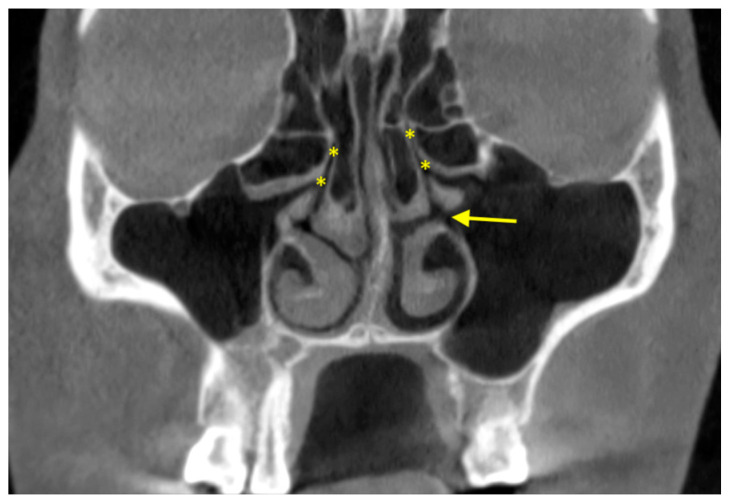
Primary ostium (asterisk) in both maxillary sinuses and accessory ostium (arrow) in the left maxillary sinus.

The sinus cavity is drained into the nasal cavity by mucociliary clearance system through the (primary) sinus ostium (Figure 3). Drainage is severed if the ostium is blocked. Ostium patency in CBCT images was found to be around 87.0–94.0% [62,63,64]. It is located above the attachment of the inferior turbinate, and most of the time it was located in the middle third (76.1%) of the turbinate or the anterior third (23.7%) [64]. Most sinus ostia were reported to be slit shaped (71.1%), followed by ovoloid (22.3%) and round (6.6%) [64]. Obstruction was significantly more likely to occur if the sinus had sinusitis or >10 mm membrane thickening [62,64]. Immediately after sinus floor elevation, it was estimated that obstruction occurred for up to 30% of the sinus ostia, which were mostly resolved at 7.5-month follow-up [63].

Meanwhile, the prevalence of accessory ostium was 35.5–56.7% [64,65,66,67]. Contrast to primary ostium, the accessory ostium was usually ovaloid (48.4%) or round (39.0%) instead of slit shaped (12.6%) [65]. The accessory ostium, if present, was on average 19.9 ± 1.7 mm from the most inferior point of the sinus floor [67]. The presence of sinusitis or mucosal thickening and accessory ostium were associated [66,67].

## 3. Pathology

### 3.1. Membrane Thickening Associated with Periapical Lesions

**Figure 4:**
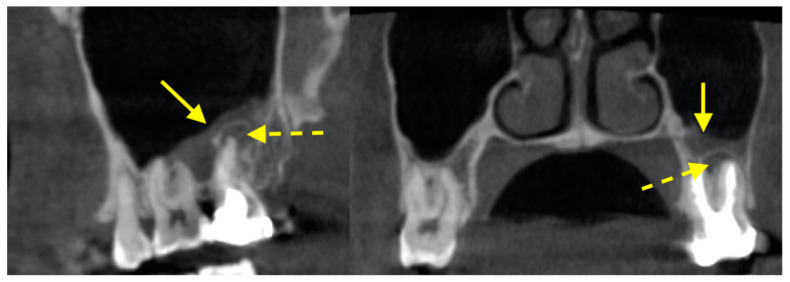
Sinus membrane thickening (solid arrow) in the left maxillary sinus associated with a periapical lesion (dotted arrow).

Sinus membrane thickening associated with an endodontically involved tooth was common (Figure 4). It has been reported by numerous studies [68,69,70,71]. In one study, the sinus membrane was, on average, 2.56–2.74 mm thick in cases with associated teeth having apical pathology, which was significantly thicker than the healthy controls (1.18–1.21 mm) [70]. In another study, the prevalence of membrane thickening was reported to be 41.5% for patients with no associated teeth with apical periodontitis, but increased to around 70% for those with visible small changes in bone structure to a well-defined radiolucent periapical area, and further increased to 100% for those with severe apical periodontitis [68]. Results from a more recent study were consistent to prior findings, in that sinuses with >2 mm membrane thickening were more likely to have associated teeth with periapical lesions (53.6% versus 42.1%) [69]. The chance of having membrane thickening when there was associated periapical lesions was estimated to be as high as thrice than when there was no periapical lesion [71]. The periapical lesion did not necessarily have to be in contact or enter the sinus floor to elicit sinus membrane thickening [68]. The number of associated root canal treatments also did not influence the degree of thickening [69].

### 3.2. Membrane Thickening Associated with Periodontal Lesions

Similar to periapical lesions, it seemed that membrane thickening was more prevalent when the associated teeth has more severe periodontal bone loss. The chance of membrane thickening for those with severe periodontal bone loss was 4.6 times than those with mild bone loss [72]. Those with class II or class III furcation lesions were 1.6 and 3.5 times, respectively, more likely to have membrane thickening than those without furcation involvement [72]. The presence of vertical intrabony pockets also led to membrane thickening being 2.4–5.6 times more likely than without such pockets [72]. The extraction of the periodontally involved tooth would lead to the complete resolution of the membrane thickening at 4 months [73]. For a comprehensive review on the effect of periapical and periodontal pathologies on membrane thickening, readers can refer to [74].

### 3.3. Mucous Retention Cyst (MRC)

**Figure 5:**
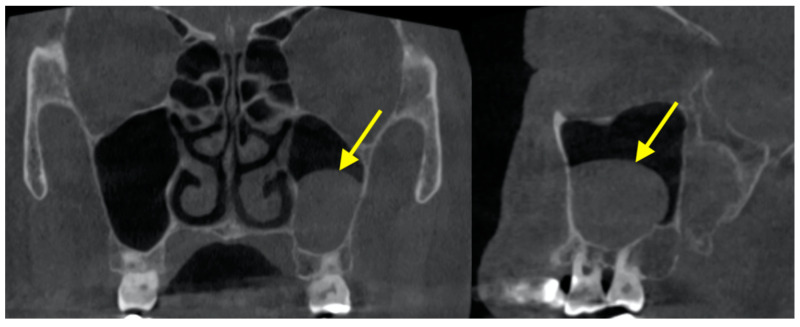
Mucous retention cyst in the left maxillary sinus.

MRCs are commonly found in the maxillary sinus (Figure 5). MRCs were found in 10.1– 28.6% of the maxillary sinus [28,75,76,77]. Up to four MRCs were reported within a single sinus [75,77]. MRCs were more commonly located at the sinus walls (19.9–53.6%) and the floor (28.6–76.7%) instead of the roof (3.4–17.9%) [75,77]. The average size of an MRC was reported to be 551 ± 1368 mm^3^ with a diameter of 9.6 ± 5.4 mm [76]. The presence of MRCs on the sinus floor was more likely if there were teeth with periapical lesions, were endodontically treated, or with severe bone loss [76,77].

### 3.4. Antrolith

**Figure 6:**
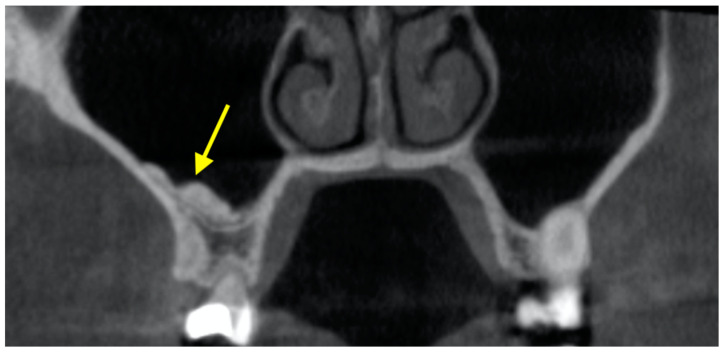
Antrolith found on the sinus floor of the right maxillary sinus.

Antrolith is a calcified mass in the maxillary sinus (Figure 6). Antroliths were found in 0.6–8.4% of maxillary sinuses [42,78,79]. They could be punctate (14.3–53.1%), linear (31.4–34.3%), or amorphous (15.5–22.9%) [78,80]. Most of them were located at the molar region (95.0%) rather than the premolar region (5.0%), and located in the sinus floor (62.9–77.5%) [79,80]. The size of an antrolith was found to be between 1–91 mm^2^, as measured by the maximum cross-sectional area computed from the coronal slices [78]. Another study reported the mean length was 5.6 ± 4.4 mm, width was 4.1 ± 2.9 mm, and height was 3.5 ± 2.1 mm [79]. Intuitively, the presence of antroliths might seem to pose an increased risk of membrane perforation following implant insertion with simultaneous sinus floor elevation, but study results did not find such increased risk [81].

### 3.5. Foreign Bodies

Due to the proximity of the maxillary sinus floor with the dentoalveolar region, iatrogenic introduction of foreign bodies into the sinus can sometimes occur. The foreign bodies can be localized with the aid of CBCT imaging. For example, one study reported 27 patients with foreign bodies introduced into their maxillary sinus, including tooth fragments, complete teeth, dental implants, dental impression material, root filling material, and dental bur [82]. Ample root filling material could be pushed into the maxillary sinus through a perforated root during root canal treatment [83]. Meanwhile, it was found that most of the cases with dental implants introduced into the sinus did not receive a presurgical CBCT exam even in the presence of a severely atrophic edentulous ridge [84]. One uncommon foreign body was a metallic hand sewing needle inserted by a patient into a cavitated tooth to remove trapped food debris, which subsequently disappeared and found to be pushed into the maxillary sinus as confirmed by CBCT [85]. Orthodontic mini-screws might also intrude into the sinus particularly if the sinus floor is <6.0 mm to the alveolar ridge crest [86].

## 4. Artificial Intelligence in Sinus Diagnosis

Nowadays, there are many research works on artificial intelligence including machine learning that can help clinicians detect abnormal findings in the maxillary sinus from CBCT images [87]. To begin with, convolutional neural network (CNN) could be used to automatically segment the maxillary sinus into the bone, air and lesion [88,89], such as MRC [90] or even complete opacification [91]. CNN could also be used to classify if a maxillary sinus was healthy or with sinusitis [92], and to detect the location of the maxillary sinus floor and automatically measure the associated alveolar ridge height for implant planning [93]. In addition, maxillary sinus features, such as sinus volume or linear measurements, were proven to be useful in dental age assessment and sex identification based on CBCT images [94,95]. Meanwhile, CBCT image filter driven by artificial intelligence has been developed to enhance the visualization of the maxillary sinus anatomy for various diagnostic purposes, such as to evaluate sinus membrane thickening associated with periapical lesions [96].

## 5. Conclusions

The use of CBCT is becoming more and more common in dental practice. The maxillary sinuses can be visualized by CBCT in a medium-to-large field-of-view volume that covers the maxillofacial region. The visualization and assessment of the maxillary sinus is very important prior to procedures that take place in close proximity with the sinus floor, such as tooth extraction, implant insertion, and sinus floor elevation. Some sinus pathologies may be associated with odontogenic lesions, such as periapical diseases and periodontal bone loss. The maxillary sinus may also involve other pathologies, such as fungal infections, allergic pathologies, benign and malignant lesions, and trauma. Clinicians should be familiar with the common anatomical features and sinus pathologies, to facilitate better diagnosis and treatment planning, and promptly refer patients to the oral and maxillofacial surgeons to follow up if needed. This review briefly covered the common and clinically relevant anatomical and pathological features concerning the maxillary sinus. It was intended to increase the awareness of the clinicians about the relevance of sinus health and pathology to clinical dentistry. To recap, the following anatomical features were reviewed: PSAA and lateral wall thickness, sinus pneumatization, sinus hypoplasia, sinus augmentation, sinus septa, and primary and accessory ostia. The following pathological features were reviewed: sinus membrane thickening associated with periapical and periodontal lesions, MRC, antrolith, and foreign bodies. The latest development in artificial intelligence in sinus diagnosis was also covered. Certainly, the features covered were not exhaustive, and readers should refer to specialized oral and maxillofacial radiology textbooks for a comprehensive description of all relevant features and proper image interpretation regarding the maxillary sinus.

## Data Availability

Not applicable.
